# Community pharmacy’s role in dispensing androgens and supporting harm reduction amid current policy dilemmas

**DOI:** 10.1186/s13011-025-00636-y

**Published:** 2025-01-18

**Authors:** Timothy Piatkowski, Lkhagvadulam Ayurzana, Michelle King, Laetitia Hattingh, Sara McMillan

**Affiliations:** 1 School of Applied Psychology, Mount Gravatt Campus, Griffith University, Mount Gravatt, QLD Australia; 2 Griffith Centre for Mental Health, Griffith University, Brisbane, QLD Australia; 3 School of Pharmacy and Medical Sciences, Griffith University, Gold Coast, QLD Australia; 4 Allied Health Research, Gold Coast Hospital and Health Service, Southport, QLD 4215 Australia; 5 School of Pharmacy, The University of Queensland, Brisbane, QLD 4102 Australia

**Keywords:** Anabolic-androgenic steroids, Androgens, Harm reduction, Pharmacists, Stigma

## Abstract

**Background:**

Legitimate androgen use, such as testosterone replacement therapy, requires a legal prescription. Off-label use for reasons like wellness and aesthetics continues to grow. Recent regulatory changes in Australia aim to curb non-prescribed androgen use, potentially intensifying stigma, however seeking prescriptions through legal channels persists. This study aimed to explore community pharmacists’ knowledge, attitudes, and practices regarding individuals who use androgens.

**Methods:**

We conducted semi-structured interviews with 15 community pharmacists, to explore knowledge and experiences related to the dispensing of androgens. The data analysis followed an iterative process, developing a codebook for thematic analysis and aligning findings with relevant literature.

**Results:**

Pharmacists face challenges when meeting the needs of individuals using androgens. They often made judgments based on appearance, leading to stigmatisation and potential refusal of prescription supply. However, this is tempered by the complex environment that pharmacists work in with respect to professional and legal requirements. Limited knowledge about androgens and varying exposure to people who use androgens were evident, prompting a unanimous desire for tailored training, especially in communication skills and interaction strategies.

**Conclusions:**

Facilitating androgen use within regulated healthcare settings, under professional medical supervision, is crucial to mitigating health risks. Varied pharmacist knowledge underscores the urgent need for targeted training, emphasising education initiatives to address structural stigma and inform healthcare policies globally.

**Supplementary Information:**

The online version contains supplementary material available at 10.1186/s13011-025-00636-y.

## Introduction

The diffusion of anabolic-androgenic steroids (AAS) from elite sports to general gym settings has resulted in diverse sub-groups of consumers which have varied motivations [1–3], risk-taking behaviour [4, 5], and engagement with healthcare services and providers [6–10]. AAS, also known as androgens, encompass testosterone and its synthetic derivatives which require a legal prescription for medical purposes [11, 12]. Testosterone replacement therapy (TRT) is indicated for organic hypogonadism resulting from pathological disorders affecting the hypothalamo-pituitary testicular axis [13, 14] and medical treatment for transgender men [15, 16]. However, there is growing concern about the lack of treatment for AAS use-induced hypogonadism [17], which may present similarly but is often not given the same clinical attention. TRT is justified when irreversible defects in the reproductive system impede androgen-sensitive tissue functions [12, 14]. The diagnostic process involves clinical assessments, hormonal assays, and a reproductive hormone profile to determine the need for lifelong testosterone replacement [13]. However, androgens are often used for off-label indications, with or without a prescription for a variety of reasons including wellness, aesthetics, and management of libido and depression [18–20].

Within the last decade a significant global surge in testosterone prescribing occurred without corresponding new approved indications [21, 22]. In Australia, this increase was primarily driven by prescriptions for currently unapproved indications for middle-aged men without reproductive pathology [21] and, thus, raised concerns about cardiovascular safety and unproven efficacy in this context [21, 23, 24]. Subsequently, the Australian Pharmaceutical Benefits Scheme (PBS) criteria were tightened in 2015 to restrict prescribing to pathology confirming low testosterone [21]. The PBS subsidises medicines based on safety, efficacy, and cost effectiveness. For men aged 18–40 years, low testosterone is defined as a serum testosterone level below 8 nmol/L, while for men aged 40–49 years, the threshold is 8nmol/L, or 8–15 nmol/L alongside other symptoms are often used as diagnostic criteria [25]​. These age-dependent thresholds reflect the natural decline in testosterone levels as men age. This context is important, as it highlights the variability in testosterone levels across different age groups, which influences eligibility for treatment under the PBS guidelines. Research evaluating the three-year effects of the tightened PBS criteria revealed decreased off-label prescriptions, yet total testosterone prescribing persisted with minimal change, suggesting a transition to private (non-PBS) prescribing [21]. These changes have led to prescribers exercising caution when initiating testosterone treatment in men without reproductive pathology [6, 10], with reports of prescribers being monitored [26] alongside claims of misconduct [27]. Subsequently, people who use androgens have reported difficulty in acquiring these medications [28, 29]. These trends map onto extant work which has documented the utilisation of androgens and other AAS for self-administered TRT [20, 30]. Individuals have reportedly engaged in self-medicating with AAS for several reasons; they did not meet the criteria for a low testosterone diagnosis, met the criteria but declined physician-recommended treatment, lacked trust in physicians thereby avoiding medical attention for low testosterone symptoms, or perceived black-market (‘underground lab’) testosterone as more cost-effective and readily available [20, 30].

As people who use AAS increasingly represent a significant portion of attendees at needle service providers, there is a growing need to integrate these harm reduction strategies into existing health services, which are currently ill-equipped to meet the unique needs of people who use AAS [31, 32]. However, there is significant stigma associated with non-prescribed androgen and illicit AAS use [33]. This stigma often complicates access to harm reduction services for those using androgens for enhancement and wellbeing purposes, particularly as harm reduction strategies for AAS, are still in their infancy. While harm reduction approaches have become foundational in the management of other substances, such as opioids [34–37], they remain underdeveloped and contentious in the context of androgens and AAS [7, 38]. This is an area in urgent need of attention given that illicitly sourced AAS often contain unknown or harmful substances [29, 39, 40], with research indicating that more than two-thirds of these products are of questionable quality [41], exposing people to heightened health risks such as increased instance of bacterial infection [29, 42]. One aspect of harm reduction for people who use AAS involves improving access to legitimate, prescription-based androgens. In Australia, pharmacists are not currently permitted to prescribe androgens [43], but they can dispense prescription medications under a doctor’s guidance.

It is important to distinguish between prescribing aimed at restoring therapeutic testosterone levels, such as in TRT for medically diagnosed hypogonadism, and the prescribing or use of androgens to achieve supra-therapeutic levels for enhancement purposes [22, 26, 44]. The former aligns with clinical guidelines and involves restoring normal physiological function, while the latter falls outside approved indications and presents heightened risks due to potential adverse effects associated with prolonged use at elevated doses. However, improving access to all types of prescribing could mitigate the significant risks associated with unregulated AAS use. Research shows that monitoring and supervising testosterone use, as opposed to unregulated and illicit AAS consumption, has yielded highly beneficial outcomes for people using AAS [45, 46]. For example, supervised testosterone administration through healthcare providers allows for regular health monitoring, such as blood tests to assess liver enzymes and blood pressure [45]. These practices help identify early warning signs of adverse health outcomes, providing a basis for informed decision-making and safer usage. Therefore, steering people who use AAS toward obtaining these substances through prescription channels, where dosages, efficacy, and safety can be better monitored, presents a potential harm reduction measure. The challenges reported by those seeking prescriptions [28] highlights the need for further examination of the factors contributing to the ongoing demand for androgens and potential consequences of all stakeholders associated with obtaining them through legal means.

Thus far, research has captured the perspectives of people who use androgens as well as healthcare providers such as medical practitioners [47–49]. The role of other healthcare providers such as community pharmacists (CPs) who not only dispense androgens but may also be requested to supply safe injecting equipment, is relatively unexplored [50]. In Australia, CPs play a pivotal role in ensuring the safe and responsible dispensing of medications, which requires careful consideration of prescriptions in compliance with established regulations and professional practice guidelines [43]. These requirements include verification of therapeutic appropriateness during the dispensing process. While a core role of CPs is dispensing valid prescriptions, they also retain professional discretion to refuse if there are concerns about safety, inappropriate prescribing, or misuse. CPs play a critical role in the provision of opioid substitution therapy for people with opioid dependence, including medications such as methadone and buprenorphine [51]. In Australia, pharmacists are often the primary point of access for these treatments, and their responsibilities extend beyond dispensing to include supervised dosing, record-keeping, and monitoring patient adherence [51, 52]. Regulations require CPs to ensure these medications are used safely and as prescribed, often involving direct communication with prescribers to manage potential risks, such as diversion [53]. This framework reflects a broader emphasis on harm reduction and patient-centred care in opioid treatment programs, highlighting the central role CPs play in supporting vulnerable populations. Despite their established role in dispensing opioid substitution medications, CPs’ engagement with people who use androgens remains underexplored. Unlike the evidence-based harm reduction frameworks available for people who use opioids, no established recommendations exist for AAS, leaving consumers with significant barriers to accessing appropriate care [10]. Therefore, the current study sought to explore CPs’ knowledge, attitudes, and practices towards people who use androgens in Australia. Given the complex structural elements which play a role in the relationships between people who use medicines and healthcare professionals [54], we draw on stigma theory to contextualise this research.

### Theoretical framing

The concept of structural stigma was applied in this study as it provides a robust framework for understanding how societal conditions, institutional policies, and cultural norms intersect to create barriers to care. This is particularly relevant given prior research indicating that people who use AAS, whether prescribed or non-prescribed, frequently report experiencing stigma from a range of healthcare providers [10, 28, 48, 55–57]. This stigma not only influences their healthcare-seeking behaviours but also exacerbates health risks by limiting access to harm reduction services or appropriate care.

Stigma theory, particularly as it relates to drug use, emphasises the dynamic and multi-level nature of stigma, spanning individual, interpersonal, and structural dimensions [58–61]. Structural stigma [62, 63], in particular, pertains to societal conditions and institutional frameworks that systematically disadvantage stigmatised groups [64–66]. This framework is highly relevant for examining healthcare contexts where societal and professional biases may shape interactions, access to services, and treatment outcomes. In the context of drug use and viral hepatitis, scholars have highlighted how structural stigma manifests through socio-political dynamics and institutional policies, creating oppressive conditions for individuals seeking care [67–70]. These insights extend to the experiences of people who inject drugs, revealing how stigma embedded in policy and practice contributes to social suffering and adverse health outcomes [71–74]. Similarly, people who use AAS—who are often excluded from mainstream harm reduction narratives—are uniquely positioned within these dynamics [1, 2], facing intersecting stigmas tied to both drug use and perceptions of their motivations for enhancement or wellbeing [33].

Given that pharmacists are key gatekeepers in healthcare provision, particularly in dispensing medications and offering harm reduction advice, understanding the ways stigma shapes their interactions with people who use AAS is critical. While structural stigma has often been used to explore the experiences of people who use drugs [50, 73–75], its application here focuses on the structural and institutional dimensions that shape pharmacists’ attitudes, knowledge, and practices. This approach allows us to examine how stigma as a form of symbolic power [66] operates within the pharmacy setting, potentially influencing care delivery and access for people who use AAS. By grounding this study in the structural stigma framework, we aim to contextualise pharmacist perspectives within the broader social, cultural, and policy environments that influence their interactions with people who use AAS. This builds on existing research to highlight the systemic and structural barriers to equitable care, extending stigma theory to a population and healthcare context that has been largely overlooked.

## Methods

### Design and ethics

This study followed the Consolidated Criteria for Reporting Qualitative research (COREQ) checklist found in the supplemental materials (see Appendix A). This study was part of a larger project which incorporated interviews with both AAS consumers and community pharmacists [76]. Ethical approval was gained from the Griffith University Human Research Ethics Committee (Approval number: 2022/794).

### Sampling and recruitment

Study participants met the inclusion criteria of being a CP in Australia and previous experience with people who use androgens. In Australia, becoming a qualified pharmacist involves completing a rigorous educational pathway, including an accredited university degree in pharmacy, a supervised internship, and passing the registration exam. Recruitment involved snowball sampling by leveraging personal and professional networks, including connections with community pharmacies and the Pharmacy Guild, as well as utilising social media platforms. The recruitment process began by reaching out to these networks and expanded iteratively through referrals. Once these avenues were exhausted, a total of 15 participants were recruited, at which point the team determined that sufficient data had been collected to capture diverse perspectives. Invited participants received a plain language statement and the option to participate or decline. Those who agreed underwent individually scheduled interviews, providing recorded verbal consent. Participants were informed of their right to withdraw at any point. A $40 gift card served as a token of appreciation for interview participation.

### Data collection

All interviews were conducted by a single interviewer who was a final year pharmacy student. The interview guide, developed by the research team and informed by the literature, followed a semi-structured format. Beginning with demographic questions, it proceeded to explore participants’ knowledge of AAS, their experiences with people who use these substances and involvement in harm reduction interventions. Example questions include: Please describe how you currently interact with AAS consumers? How confident are you in terms of what AAS consumers require from your service? Could you tell me a bit more about what AAS consumers seek from you in terms of service/s? and could you give me an example? To validate the interview questions and enhance the researcher’s confidence, four pilot interviews were conducted with pharmacists, two of whom were from the research team. Minimal changes were subsequently made to the flow of questions. Interviews were scheduled based on participant availability and conducted on the Microsoft Teams platform to be automatically transcribed. Debriefs were provided to the research team after each interview to facilitate further data discussion. To ensure accuracy, the interviewer manually reviewed the transcriptions, and the data were subsequently imported into NVivo (QSR, v12), a qualitative data analysis software, for analysis. Transcriptions were returned to two participants as requested but no changes made.

### Data analysis

This study employed an iterative and collaborative process for developing a codebook, drawing on methodologies outlined by Neale [77]. Initially, deductive codes were formulated based on the predetermined topics of interest outlined in the interview guide, such as “knowledge” and “patient-pharmacist relationship.” Additionally, inductive codes, including “stigma,” and “structural stigma” were generated through ongoing team discussions addressing emerging themes. Various iterations of the codebook were tested on transcript excerpts to establish interpretive consensus [78]. Subsequently, a single coder utilised NVivo to apply the finalised codes to all interview transcripts. The analysis focused on reviewing codes related to specific topics (e.g., “stigma,” “knowledge gaps”) to identify emergent analytic themes (e.g., “structural stigma” “engagement,” “stereotypes”). Themes were documented through memos, fostering regular discussions within the research team. These discussions were robust, based on the different academic and clinical backgrounds of the research team, which included expertise in pharmacy law and ethics, pharmacy practice, and drug policy and harm reduction practice regarding AAS. The analysis was conducted in conjunction with a comprehensive review of relevant literature, with particular attention to aspects of stigma as defined in the theoretical framework.

## Results

Fifteen participants (8 females, 7 males) aged 25 to 49 years (*Mean* = 33, *SD* = 7.16) were interviewed between July to August 2023. All the participants were working in a community pharmacy at the time of the interview and had on average 10 years of community experience. Most participants were from Queensland, Australia (*n* = 13) and two participants were from New South Wales. Three participants worked in regional areas. In terms of community pharmacy type, five participants were employed in independent pharmacies whereas the remaining 10 participants worked in banner group pharmacies. The mean interview duration was approximately 30 min (*SD* = 5.27).

### Systems contributing to structural stigma: “I might tell a little fib and say ‘ohh, we don’t have any in stock’”

Pharmacists articulated a sense of heightened vigilance when interacting with individuals ‘using substances,’ with some hesitancy in supplying AAS reflecting some underlying biases and stigmatisation among CPs, alongside questions about professional responsibility. Mostly, CPs perceived people who used androgens as predominantly young males exhibiting a muscular physique. In the literature this type of behaviour has been referred to as ‘muscle profiling’ [79] and leads to assumptions and stereotyping. However, we acknowledge that in the pharmacy environment these types of evaluations are necessary and made in the interest of maintaining the health and wellbeing of the patient. In these instances, however, judgments made about aesthetic motivations underlying the prescription contribute to overlooking the potential therapeutic need based on external appearances. Such stereotyping can influence pharmacist-consumer interactions and relationships and contribute to the broader issue of stigmatisation associated with both AAS [10] and substance use more broadly [59].

*P3: I guess the TGA [Therapeutic Goods Administration] registered indication is for people with the hormonal issues underperforming like there’s lower than baseline testosterone etcetera*,* usually you see. I guess. The population would not be young*,* athletic men. So*,* and that this is just from my visual observation*,* the guys that come in and they’re muscly they’re very lean… it doesn’t take a rocket scientist to know it is for them for aesthetic purposes.*

While some pharmacists acknowledged the potential legitimacy of use for medical reasons, there was a tendency among participants to make quick judgments about the probability of inappropriate use based on appearance.

*P8: Sometimes we use our judgement of*,* you know*,* age and physique and so forth. And I guess that’s probably a rush generalisation. If they’re quite stocky and they look like they go to the gym quite regularly*,* that doesn’t mean to say that they don’t have other conditions that they could be legitimately taking the medication for….*

*P15: I think we judge them a little bit too early with that. Personally*,* I think there may be some customers who just don’t fit the criteria. They’re using it for body enhancement.*

Despite recognising the limitations of these judgments, CPs expressed concerns about people who use androgens not fitting predetermined evidence-based therapeutic criteria or using substances for body enhancement. This underscores a complex interplay between individual judgments and preconceived notions about the ‘right’ reasons for using substances [58], and the pharmacists’ Code of Ethics that requires pharmacists to consider patients’ wellbeing as well as autonomy [80]. Ultimately, the discussions from pharmacists speak to a degree of structural issues which may be embedded in healthcare settings [81]. Even if individuals attempt to use substances for approved medical purposes, they may still face scrutiny, reflecting the broader issue of stigma in healthcare encounters for people who use drugs [73, 81].

*P4: …the patient was in his early twenties*,* and he was already quite muscular*,* and he had a prescription for you know*,* testosterone from a doctor and I guess that*,* I mean*,* you know the chances of him actually having low testosterone just based on his appearance*,* did seem quite low.*

*P6: But when you see a certain individual who is quite robust and muscular and what not coming in for testosterone or HGH [human growth hormone] or whatever it might be… that’s when you sort of can go like ‘okay*,* these aren’t realistically being used for the purposes that are intended for’.*

Some CPs described engaging in discussions with people who use androgens about their usage, an important positive step toward building a therapeutic relationship and understanding their needs. However, an underlying level of scepticism was evident in some descriptions, indicating a potential stigmatising lens through which these discussions occur.

*P8: If I do receive a prescription for something like this [androgens]*,* I always consult with the patient to see what they’re actually using it for. Sometimes you might get dubious answers or incorrect information*,* so generally if they’re using it for a condition which has been approved for use*,* they will open up that information.*

Whilst therapeutic need is required to be established for all medicines, it is evident that there is increased attention given to medicines that could be used for off-label reasons. When asking about use, one CP observed a shift in the responses from individuals who used androgens, noting an increased sophistication in the narratives provided. Patients seemed to provide CPs with more detailed explanations, citing factors like low hormone levels and interactions with endocrinologists:

*P9: … [They’ve] Levelled up*,* they’ll make up*,* or they’ll have a whole like back story now. So*,* they’ll have like “ohh my levels are really low then this range. I see my endo [endocrinologist]; you know and this time I don’t meet the requirements to get a PBS [Pharmaceutical Benefits Scheme] [prescription].”*

This response may stem from the perceived stigma associated with AAS use, aligning justification with the therapeutic reasons for which they are approved to be prescribed [11, 82]. The pharmacist’s characterisation of these narratives as being “made up” suggests a lens influenced by stigma and judgement [59]. The physical appearance of individuals and prescriptions being non-PBS (private) also prompted pharmacists to question the legitimacy of prescriptions.

*P10: I guess my assumption would be mainly based around the prescription being a private prescription from the GP [general practitioner] and then mainly based on the physical appearance of the people coming to collect the medication.*

Limited prescriber and pharmacist collaboration or information about doctor-patient consultations added to a level of scepticism about prescription legitimacy. This lack of insight in the doctor-patient relationship may result in a reluctance to provide healthcare services and necessary harm reduction support, thereby perpetuating the cycle of structural bias against individuals seeking androgens.

*P5: If they’ve gotten authority script*,* then I guess supposedly the doctor must have done a blood test or that maybe they’ve been threatened in a little meeting room. I don’t know.*

Some pharmacists admitted to a sense of fear toward this population, attributing it to their perceived ‘intimidating’ and ‘robust’ physical presence. A couple of pharmacists described some altercations related to medication refusal, these concerns may be influenced by societal narratives, often perpetuated by media portrayals, associating people who use AAS with aggression and violence [28, 83, 84]. Despite the lack of empirical evidence supporting this stereotype [85], the pharmacists’ concerns about potential verbal and physical altercations highlight the impact of these narratives on the perceptions which healthcare professional have of those seeking androgens.

*P8: I think there’s*,* you know*,* that fear*,* that perception*,* that these patients can become quite aggressive if you do refuse supply. You always worry about copping some verbal abuse or even physical abuse or*,* you know*,* they might damage the shop in some ways in terms of*,* you know*,* like throwing products or breaking something*,* you know you don’t know where it’s going to escalate.*

*P9: You’re a little bit on edge because I feel like those kinds of people*,* you don’t know how they’re going to react. A lot of them are really under influence or*,* you know*,* might be having roid rage or something. So*,* you*,* you’re a bit hesitant to deal with those people as you would with*,* you know*,* other*,* you know*,* patients or consumers that come into the pharmacy.*

Some pharmacists disclosed providing false information about product availability or claiming unavailability to avoid confrontation from customers seeking specific substances. Another pharmacist described store policy of refusing to dispense any AAS private prescriptions. These practices reflect some broader systemic issues for those working in community pharmacy. Concerns for consumer health and safety influence pharmacists’ decisions are made more challenging by systemic issues related to prescribing [11] and the illicit nature of non-prescribed androgens in Australia [28]. Therefore, it is essential to balance these considerations with a commitment to ensuring equitable access to healthcare for this population.

*P6: …then I might*,* you know*,* I might tell a little fib and say like “ohh*,* we don’t have any in stock” or whatever else like that and just sort of do to sort of move them on that way.*

*P12: If the men have a private script for Primoteston [testosterone] unfortunately*,* to avoid anger I often just say “I don’t have it” and at the moment we don’t because we can’t get it*,* so that’s good… it’s unfortunate that sometimes I choose to lie to a person rather than confront them with the fact that I’m just not giving you something that you want.*

### Engagement and training: “I don’t feel like I am fully equipped to help”

Questions were raised by pharmacists about whether it was ‘*professionally appropriate for us to be dispensing these [legal prescriptions]’ (P3).* There have been cases of professional misconduct where pharmacists have been suspended for inappropriate dispensing of AAS [86], and this concern was in the forefront of some pharmacists:

*P8: …this was a patient who was frequently getting medication and obviously using too much of it. And he [pharmacist] did contact the doctor several times and the doctor approved the dispensing of the medication*,* and the pharmacist went ahead and dispensed the medication. And it*,* you know*,* came back to the pharmacist not exercising their professional judgement for refusing supply*,* and so they were deregistered. So that’s always at the back of my mind…*.

Despite attempts to confirm therapeutic need by contacting the prescriber, the above detail supports the notion that pharmacists, as independent health care providers, should refuse supply if they have remaining questions about the prescriber’s intention. This is an example of an ethical dilemma that pharmacists can be exposed to. There were also safety concerns about people seeking AAS from less reputable sources if they could not obtain them from a legitimate source:

*P13: …they’re ordering outside of Australia*,* it’s outside of our sort of ability to ensure that it’s safe and appropriate for them. I personally feel. It’s not the most appropriate or safe option to do…it’s not leaving any history of them taking that medication. We can’t really keep track of how they’re going with it…*.

Pharmacists were transparent in explaining that if they ask more questions and identify ‘non-therapeutic’ use then this could lead them to refusing supply, yet if not asking questions and supply, this may avoid any supportive discussions around safer use.

*P2: …Obviously*,* there is an assumption that the doctor is going to be the one injecting it*,* because that would be the correct appropriate thing. So*,* we don’t offer them a sharps kit or anything like that or discuss the materials they need unless they specifically ask because*,* you know*,* theoretically none of them should need those things because the doctor would have the materials so that can be challenging because*,* you cannot identify the people who need the counselling. They have to ask*,* and that does make it a bit more difficult. I think.*

This variability in practice emphasises the need for further professional support for pharmacist decision making, to reduce the conflict between legal and professional responsibilities whilst supporting patient autonomy and wellbeing.

The perceptions and knowledge base of CPs shaped their interactions with people using these substances. Participants who acknowledged limited understanding of the subject, expressed a hesitancy to actively seek information from people who use androgens, indicating that a perceived lack of expertise influenced their past engagement. Ultimately, this suggests that pharmacists who felt less knowledgeable about androgens may have been more cautious or reserved in their approach.

*P10: I wouldn’t feel like I was super knowledgeable in the subject anyway*,* so I would be less likely to probe for information off them because I don’t feel like I am fully equipped to help.*

There was unanimous interest expressed by participants in pursuing further training. Participants underscored a perceived gap in effective communication skills and guidance related to androgens and AAS more broadly. Therefore, CPs articulated a need for training specifically around interaction strategies, choice of language, adept questioning techniques, and appropriate responses in diverse situations involving people using androgens, emphasising a practical orientation to engagement strategies in the context of these encounters.

*P9: [On training] I guess it would just be how to interact kind of words you use. How to*,* what kind of how to ask*,* particular questions. How they respond maybe and what to do in certain situations.*

Several pharmacists expressed interest in gaining a deeper understanding of the motivations behind individuals choosing to use androgens and sought comprehensive therapeutic knowledge regarding their regimens to better assist the community. Participants highlighted a perceived gap in guidelines, particularly concerning injection regimens, such as type of substance, dosage, and frequency. The need for training extended beyond the physical injection process, with a desire for insights into the reasons for use and the associated short- and long-term effects. Interestingly, moving past provision of safe injecting information has been reflected in extant work [87] and underscores the healthcare sector’s aspiration for more holistic education which equips health professionals with the knowledge to provide comprehensive harm reduction information to people who use androgens.

*P2: I don’t know how they do them and how often to do it. I don’t know how they do these regimens. I think they are going off information online. So*,* I think that’s where that information would focus*,* is giving me the information to provide them with quality information about how to use these medications safely*,* not just how to physically inject them. That’s all I can help them with now is I can help them with how to physically inject*,* but I can’t really talk to them about how often they should be using it. How much any of that because there are no guidelines that I know of.*

*P3: [I want to know] … why people use them? You know*,* like some people might use them to bulk up. Some people use it to lose fat. It would be interesting to know. It’ll be interesting [to have a] really good dive to know about the long*,* short term and long-term complications of the substances because it would be good to tell patients regardless because you know anyone that uses would be at risk.*

CPs expressed a desire for specific training and supplementary resources to enhance their ability to identify emerging trends and novel substances within the broader AAS landscape. This sentiment is rooted in the recognition of the dynamic nature of androgen usage patterns and the need to stay abreast of evolving trends, emphasising the importance of continuous education and resources tailored to the changing landscape of androgens.

*P6: I think some specific training would be great for people just to identify trends or new molecules that are being used for certain things by different people. I’m sure as we’re all aware*,* things chop and change all the time*,* and what’s popular*,* what’s not popular*,* what’s being used*,* that’s not being used.*

## Discussion

To our knowledge, this is the first study globally to reveal the influence of broader systemic issues which affect community pharmacy setting regarding the dispensing of androgens. Our findings reveal intricate dynamics influencing the interactions between CPs and people seeking androgens through means of regulated and safe supply. Some pharmacists’ decisions were influenced by preconceived notions, leading to potential denial or questioning the supply of androgens. Concerns about potential unpredictable behaviour among individuals using androgens were in some instances due to previous negative experiences but could also highlight underlying biases which are likely caused by media propaganda and result in stigmatisation [28, 83, 84]. Stigma, particularly when reinforced by media portrayals and societal biases, can significantly impact how healthcare providers, including pharmacists, perceive and interact with people who use drugs [88, 89], as well as specifically those using androgens [33, 50]. This stigmatisation often manifests in reluctance to provide necessary care, further alienating people who use androgens and pushing them toward unregulated, riskier sources. Whilst acknowledging the complex interplay of ethical decisions to supply AAS whilst considering the wellbeing of patients, collectively, the data reveal a critical knowledge gap among CPs. Inadequate education about harm reduction principles exacerbates these biases, leading to reduced access to safe, regulated supplies of androgens. Addressing stigma through targeted training and promoting harm reduction within the healthcare system is essential to ensure equitable, nonjudgmental care that prioritises the health and safety of this population.

CPs are placed in a challenging position, which reflects systemic issues surrounding androgens in Australia. Without clear policy, healthcare services are left in a difficult position with those seeking androgen dispensing. However, AAS-related risks, while different in nature, require equitable and considered approaches which fit the needs of the group. For example, policies could mandate and support the integration of AAS-specific training for pharmacists, ensuring that professionals can respond to the distinct health and cultural needs of this group. In Australia, it is legally possible for a third party to collect prescription medications, including androgens, on behalf of someone else [43]. However, this face-to-face engagement allows pharmacists to provide advice on proper dosing, potential side effects, and safe administration, as well as to conduct health checks, which are crucial for mitigating the risks associated with androgen use. Policies could be introduced to encourage or even mandate brief health consultations at the point of dispensing, similar to the models employed in instances of opioid dispensing [90]. This would ensure that people who use androgens not only receive their medications but also benefit from health monitoring, risk minimisation education, and support for any related health concerns.

It is important to note this research comes with caveats. For instance, the use of non-prescribed AAS poses well-documented physical [91], psychological [92–94], and social harms [95, 96]. Risks include infections from injecting illicit AAS and transmission of blood-borne viruses [42]. Furthermore, long-term AAS use raises concerns about cardiovascular and liver damage [24, 97], cognitive effects [98], and the development of AAS-induced hypogonadism, impacting reproductive and mental health [99]. Despite awareness of these risks, individuals often prioritise perceived benefits, such as muscle mass retention and social status, leading to continued AAS use. This provides some important contextual factors to consider when interpreting study findings; CPs are placed in a challenging position if they have concerns about consumer wellbeing when presented with a prescription for androgens. Therefore, while acknowledging the documented physical and psychological risks associated with androgen use, it is imperative to recognise that many of these harms are compounded by structural factors inherent in illicit usage [28] sharing semblance with other illicit substances [74]. The dangers of AAS-related harms are often exacerbated when individuals resort to non-regulated, non-medical sources. Facilitating androgen use within a healthcare setting, under appropriate prescribing, dispensing and supervision by healthcare professionals, is likely to mitigate these health risks [45, 46], emphasising the importance of a regulated and supportive healthcare approach in managing androgen use in the community. Within the Australian context, the existing punitive and restrictive drug policy regarding AAS poses challenges, particularly in the absence of decriminalisation measures. As testosterone replacement remains a viable option for some people, addressing the systemic barriers is crucial.

While some might argue that enabling access to prescription androgens legitimises non-medical use, harm reduction prioritises the minimisation of adverse outcomes over moral judgment [100]. As seen in other domains of public health, harm reduction strategies have demonstrated efficacy by reducing risks without increasing consumption or creating problematic use [101] as well as a host of other social and economic benefits [102–104]. Offering safer, medically supervised options aligns with harm reduction principles, and in some contexts, this approach is referred to as safe supply prescribing [105, 106]. While not formalised in Australian policy for androgens, exploring these parallels is important to contextualising harm reduction for AAS. Offering safer, medically supervised options aligns with this principle.

Similar parallels can be drawn from the concept of safe supply prescribing in other contexts, such as the regulated supply of opioids to people with opioid use disorder. Safe supply prescribing aims to reduce the harms associated with illicit opioid use by offering safer, medically regulated alternatives that mitigate health risks while addressing underlying medical and social issues [34, 35]. While methadone and buprenorphine are established treatments for opioid use disorder [107–109], safe supply approaches focus on offering regulated, legal access to the substances themselves to reduce harm from illicit sources [110]. In both cases, the goal is not to promote drug use, but to offer regulated alternatives that mitigate health risks and provide a safer pathway for individuals. Parallels to this model have been explored in the literature [38, 105, 111–113]. In the absence of a regulated market for many of these substances, ensuring access through legitimate medical frameworks can help safeguard people who use AAS from harmful impurities or inappropriate dosages. Pharmacies, already equipped to dispense controlled substances for harm reduction purposes, are well-positioned to play a similar role in androgen use. By providing safer, medically regulated access to androgens under prescription, pharmacies can ensure proper dosing, reduce the reliance on illicit markets, and offer crucial health advice to people who use these substances.

This model of supervised and regulated supply not only minimises risks associated with contaminated or mislabelled products but can also create an environment where consumers can receive education and support [76] —services that are already effectively integrated within opioid dependence treatment frameworks [89, 114, 115]. For instance, CPs who receive proper androgen and AAS training could provide essential services such as offering accurate information on dosage, health monitoring, and potential side effects. Pharmacists are well-positioned to counsel people on appropriate dosing, explaining that higher doses may lead to toxicity and adverse outcomes without additional therapeutic benefit, thereby promoting safer usage practices. Further, by integrating blood pressure checks and ongoing health assessments into their services, pharmacists could help prevent many of the adverse effects associated with improper AAS use, much like how opioid dependence programs monitor patient health to ensure the safe use of controlled substances [116]. Additionally, pharmacists could support people who use AAS by providing education on safer injection techniques and sterile equipment, similar to needle exchange programs, reducing the risk of infections and other complications. These strategies mirror those already employed in OST, where pharmacists not only dispense medications but also provide tailored advice, health interventions, and support, making them ideal to apply similar frameworks for AAS consumers. Thus, while harm reduction frameworks underpin this discussion, aligning with the safe supply prescribing model where appropriate ensures the approach remains adaptable to different regulatory and policy contexts. Expanding pharmacist involvement in this space could transform how harm reduction is enacted for AAS consumption, offering a safer, medically supervised pathway that reduces reliance on illicit markets.

One limitation of the study is that it did not explore the ethical reasoning of pharmacists, representing an area that warrants further investigation for a more fulsome understanding of decision-making processes surrounding androgen dispensing. However, our study revealed varied knowledge among CPs regarding androgens and AAS, influenced by exposure and experience. As outlined already, CPs expressed a need for targeted training in communication and therapeutic use (e.g. differences in the action of various dosage forms), emphasising a desire for a deeper understanding of motivations, regimens, and complications related to androgen use. They welcomed continuous education to address evolving trends. The findings highlight the imperative need for education initiatives. Implementing such initiatives can enhance CPs’ competency in dispensing, communication, and understanding the complexities of androgen use. Additionally, providing targeted training and evidence-based guidance can foster greater confidence and security for CPs, equipping them to make informed decisions without fear of professional misconduct or accusations, which have been identified as key concerns. Future research should focus on developing targeted training for CPs and healthcare interventions to reduce stigma and enhance pharmacist-patient relationships, globally informing policies for unbiased healthcare and promoting better therapeutic relationships and patient outcomes. In the absence of decriminalisation, establishing supportive, non-stigmatising pathways for individuals seeking testosterone use becomes imperative to navigate the intersection of health, drug policy, and individual wellbeing within the community.

## Electronic supplementary material

Below is the link to the electronic supplementary material.

Supplementary Material 1

## Data Availability

Availability of data and materials: Available from corresponding author on reasonable request.

## Abbreviations

AAS: Anabolic-androgenic steroids
CP: Community pharmacist
TRT: Testosterone replacement therapy
