# Supplementary material for: Community pharmacy’s role in dispensing androgens and supporting harm reduction amid current policy dilemmas
Source: Subst Abuse Treat Prev Policy. 2025 Jan 18;20:2. doi: 10.1186/s13011-025-00636-y (PMC11748596; doi:10.1186/s13011-025-00636-y)
Supplement: Supplementary file 1 — Supplementary Material 1 [file 13011_2025_636_MOESM1_ESM.docx]

**Appendix A.**

### COREQ Criteria Checklist

**Developed from:**

Tong A, Sainsbury P, Craig J. Consolidated criteria for reporting qualitative research (COREQ): a 32-item checklist for interviews and focus groups. *International Journal for Quality in Health Care*. 2007. Volume 19, Number 6: pp. 349 – 357

| **No.** | **Item** | **Guide Questions/Description** | **Notes and/or Section Reported in** |
| --- | --- | --- | --- |
| **Domain 1: Research team and reﬂexivity** | | | |
| *Personal Characteristics* | | | |
| 1 | Interviewer/  facilitator | Which author/s conducted the interview or focus group? | Lkhagvadulam Ayurzana |
| 2 | Credentials | What were the researcher’s credentials? E.g. PhD, MD | The interviewer is a Master of Pharmacy Student. All other members of the research team have PhD’s. |
| 3 | Occupation | What was their occupation at the time of the study? | The interviewer is a student and is employed in community pharmacy part-time. All other members of the research team are academics. |
| 4 | Gender | Was the researcher male or female? | Lkhagvadulam Ayurzana (the interviewer) and A/Prof Hattingh, A/Prof King, and Dr McMillan are female, Dr Piatkowski is male. |
| 5 | Experience and training | What experience or training did the researcher have? | The interviewer is a Master of Pharmacy student who has been employed part-time in a community pharmacy for one year at the time of the study. All other members of the research team have doctorate level qualifications and a long history in qualitative research. |
| *Relationship with participants* | | | |
| 6 | Relationship established | Was a relationship established prior to study commencement? | No. Initial contact was only to establish interest in the study by providing the Participant Information Sheet and to organise a time for interview. |
| 7 | Participant knowledge of the interviewer | What did the participants know about the researcher? e.g. personal goals, reasons for doing the research | Only information provided in the Participant Information Sheet and Interview Guide. See Method section and Interview Guide. |
| 8 | Interviewer characteristics | What characteristics were reported about the interviewer/facilitator? e.g. Bias, assumptions, reasons and interests in the research topic | Only information provided in the Participant Information Sheet and Interview Guide. See Method section and Interview Guide. |
| **Domain 2: Study design** | | | |
| *Theoretical framework* | | | |
| 9 | Methodological orientation, ontological or epistemological basis | What methodological orientation was stated to underpin the study? e.g. grounded theory, discourse analysis, ethnography, phenomenology, content analysis | Pilot study, exploratory in nature. See Method section for more information. |
| *Participant selection* | | | |
| 10 | Sampling | How were participants selected? e.g. purposive, convenience, consecutive, snowball | Purposive and snowball sampling. See Method section for more information. |
| 11 | Method of approach | How were participants approached? e.g. face-to-face, telephone, mail, email | Described in the Method section. |
| 12 | Sample size | How many participants were in the study? | A total of 15 community pharmacists agreed to participate in this study. |
| 13 | Non-participation | How many people refused to participate or dropped out? Reasons? | 15 interviews were conducted. See Methods section for more information. |
| 14 | Setting of data collection | Where was the data collected? e.g. home, clinic, workplace | Collection of data was via the recording of a Microsoft Teams interview with participants. The researcher recommended participants to be a in quiet place free from distractions during the duration of the interview. |
| 15 | Presence of non-participants | Was anyone else present besides the participants and researchers? | Unable to be determined as interviews were conducted over Teams. No other persons were present on the researcher’s end. |
| 16 | Description of sample | What are the important characteristics of the sample? e.g. demographic data, date | Refer to Results. |
| *Data collection* | | | |
| 17 | Interview guide | Were questions, prompts, guides provided by the authors? Was it pilot tested? | Refer to Methods. The Interview Guide was pilot tested through the use of mock interviews with members of the research team and community pharmacists. Adjustments were made to the Interview Guide after the first participant interview. See Method section for more information. |
| 18 | Repeat interviews | Were repeat interviews carried out? If yes, how many? | No repeat interviews were conducted with participants. |
| 19 | Audio/visual recording | Did the research use audio or visual recording to collect the data? | Audio recording only. See Method section for more information. |
| 20 | Field notes | Were ﬁeld notes made during and/or after the interview or focus group? | Field notes were taken during and after the interviews. See Method section for more information. |
| 21 | Duration | What was the duration of the interviews or focus group? | 30-60-minute interviews. |
| 22 | Data saturation | Was data saturation discussed? | Yes. See Method section. |
| 23 | Transcripts returned | Were transcripts returned to participants for comment and/or correction? | Transcripts were offered to participants for review. Two participants took up the offer. |
| **Domain 3: Analysis and findings** | | | |
| *Data analysis* | | | |
| 24 | Number of data coders | How many data coders coded the data? | One data coder coded the data in collaboration with the whole research team. |
| 25 | Description of the coding tree | Did authors provide a description of the coding tree? | Yes. See Method section. |
| 26 | Derivation of themes | Were themes identiﬁed in advance or derived from the data? | Themes were derived from the data. See Method section on the process. |
| 27 | Software | What software, if applicable, was used to manage the data? | Qualitative data analysis software NVivo Version 12 was used to organise the interview transcripts. |
| 28 | Participant checking | Did participants provide feedback on the ﬁndings? | No, but copies of the findings were provided to all participants. |
| *Reporting* | | | |
| 29 | Quotations presented | Were participant quotations presented to illustrate the themes/ﬁndings? Was each quotation identiﬁed? e.g. participant number | Yes. See Findings section. |
| 30 | Data and ﬁndings consistent | Was there consistency between the data presented and the ﬁndings? | Yes, findings were directly derived from the data. See Findings and Discussion sections for more information. |
| 31 | Clarity of major themes | Were major themes clearly presented in the ﬁndings? | Yes. Themes were discussed in the Findings section. See Findings section for more information. |
| 32 | Clarity of minor themes | Is there a description of diverse cases or discussion of minor themes? | Data was highly homogenous. Minor discussion of diverse cases occurs in the Findings section. |
